# RNAseq-Based Prioritization Revealed *COL6A5*, *COL8A1*, *COL10A1* and *MIR146A* as Common and Differential Susceptibility Biomarkers for Psoriasis and Psoriatic Arthritis: Confirmation from Genotyping Analysis of 1417 Italian Subjects

**DOI:** 10.3390/ijms21082740

**Published:** 2020-04-15

**Authors:** Valerio Caputo, Claudia Strafella, Andrea Termine, Elena Campione, Luca Bianchi, Giuseppe Novelli, Emiliano Giardina, Raffaella Cascella

**Affiliations:** 1 Medical Genetics Laboratory, Department of Biomedicine and Prevention, Tor Vergata University, 00133 Rome, Italy; v.caputo91@gmail.com (V.C.); claudia.strafella@gmail.com (C.S.); gnovelli@me.com (G.N.); emiliano.giardina@uniroma2.it (E.G.); 2 Genomic Medicine Laboratory UILDM, Santa Lucia Foundation, 00179 Rome, Italy; andreatermine544@gmail.com; 3 Dermatologic Clinic, Department of Systems Medicine, Tor Vergata University, 00133 Rome, Italy; campioneelena@hotmail.com (E.C.); luca.bianchi@uniroma2.it (L.B.); 4 Neuromed Institute IRCCS, 86077 Pozzilli, Italy; 5 Department of Biomedical Sciences, Catholic University Our Lady of Good Counsel, 1000 Tirana, Albania

**Keywords:** genomic analysis, bioinformatics tools, psoriasis, psoriatic arthritis, collagens, biomarkers

## Abstract

Psoriasis (Ps) and Psoriatic Arthritis (PsA) are characterized by a multifactorial etiology, involving genetic and environmental factors. The present study aimed to investigate polymorphisms (SNPs) within genes involved in extracellular matrix and cell homeostasis and microRNA genes as susceptibility biomarkers for Ps and PsA. Bioinformatic analysis on public RNA-seq data allowed for selection of rs12488457 (A/C, *COL6A5*), rs13081855 (G/T, *COL8A1*), rs3812111 (A/T, *COL10A1*) and rs2910164 (C/G, *MIR146A*) as candidate biomarkers. These polymorphisms were analyzed by Real-Time PCR in a cohort of 1417 Italian patients (393 Ps, 424 PsA, 600 controls). Statistical and bioinformatic tools were utilized for assessing the genetic association and predicting the effects of the selected SNPs. rs12488457, rs13081855 and rs2910164 were significantly associated with both Ps (*p* = 1.39 × 10^−8^, *p* = 4.52 × 10^−4^, *p* = 0.04, respectively) and PsA (*p* = 5.12 × 10^−5^, *p* = 1.19 × 10^−6^, *p* = 0.01, respectively). rs3812111, instead, was associated only with PsA (*p* = 0.005). Bioinformatic analysis revealed common and differential biological pathways involved in Ps and PsA. *COL6A5* and *COL8A1* take part in the proliferation and angiogenic pathways which are altered in Ps/PsA and contribute to inflammation together with *MIR146A*. On the other hand, the exclusive association of *COL10A1* with PsA highlighted the specific involvement of bone metabolism in PsA.

## 1. Introduction

Psoriasis (Ps) and Psoriatic Arthritis (PsA) are chronic, inflammatory, immune-mediated pathologies characterized by a multifactorial etiology [1,2]. Both disorders are highly heterogeneous from a clinical point of view. Psoriatic skin lesions commonly appear on the scalp, elbows, knees, and lumbar area, and are responsible for itching, stinging and painful reactions [3]. The onset of PsA, instead, is generally characterized by arthritis, enthesitis, dactylitis and synovitis combined with the aforementioned psoriatic cutaneous manifestations [4,5].

Ps can affect people of all ages and occurs with a variable prevalence ranging from 0.09% to 11.43%, depending on the geographic area. Approximately 20–30% of Ps patients develop PsA during their lifetime. The etiopathogenesis of Ps and PsA involves both genetic, epigenetic and nongenetic factors. In particular, smoking, infections, drugs and obesity are common susceptibility factors [3]. Moreover, several genes and variants have been associated with a higher risk to develop Ps and/or PsA and most of them are shared between them (e.g., *HLA-C*, *TRAF3IP2*, *IL12B*, *IL23A*, *IL23R*, *TYK2*, *LCE3B*, *LCE3C*, *REL*, *TNIP1* and *FBXL19*) [6]. However, several studies have reported the implication of genes and variants specifically associated with PsA, suggesting the existence of differential molecular pathways underlying the onset and progression of Ps and PsA. For instance, polymorphisms located within *Interleukin 4* (*IL4*, 5q31) and *Kinesin Family Member 3A* (*KIF3A*, 5q31) genes have been found exclusively associated with PsA and are known to be specifically implicated in bone metabolism [7]. Furthermore, epigenetics have been supposed to play an important role in increasing the susceptibility to Ps and PsA. In particular, different microRNAs (miRNAs) have been involved in the development of both disorders and variants located within genes encoding miRNAs have been investigated as potential disease contributors [8,9].

The identification of common and differential biomarkers for Ps and PsA can be relevant for dissecting the pathogenetic mechanisms leading to the development of such disorders and, eventually, setting up novel and tailored treatments. In this context, investigating the role of the extracellular matrix (ECM) and its components may provide interesting insights into the etiopathogenetic pathways leading to the onset and progression of Ps and PsA. The most abundant components of the ECM are collagens, the fibrous proteins that are involved in the formation and maintenance of structural components and provide mechanical strength and elasticity to connective tissues (such as cartilage, bone, epidermis, cornea and blood vessels) [10,11].

The present study aimed to analyze single nucleotide polymorphisms (SNPs) within genes involved in ECM maintenance and cell homeostasis. In particular, a prioritization analysis was performed to select the genes of interest, taking into account the gene expression profiles from RNA sequencing (RNA-seq) data of psoriatic and healthy skin samples available from the Gene Expression Omnibus (GEO) database (https://www.ncbi.nlm.nih.gov/gds). Among the Differentially Expressed Genes (DEGs) of the RNA-seq dataset, collagen genes were extracted. Subsequently, we performed a Gene Set Enrichment Analysis (GSEA) to investigate the molecular pathways in which collagen genes may be involved. Therefore, we prioritized *Collagen, Type X, Alpha-1*(*COL10A1*, 6q22.1), *Collagen, Type VI, Alpha-5* (*COL6A5*, 3q22.1), *Collagen, Type VIII*, and *Alpha-1*(*COL8A1*, 3q12.1), since they have been involved in ECM-cell interactions, cell proliferation, regulation of angiogenesis, and bone morphogenesis [6,7].

Moreover, polymorphisms within these genes have been reported to contribute to the generation of abnormal connective tissue [12]. Given these preliminary data, we selected three SNPs within the prioritized genes to be tested as Ps/PsA susceptibility biomarkers, namely rs3812111 (A/T, *COL10A1*), rs12488457 (A/C, *COL6A5*) and rs13081855 (G/T, *COL8A1*). In particular, we selected these SNPs considering the literature and our own laboratory works, which have previously investigated them as susceptibility factors for Age-related Macular Degeneration (AMD) and Atopic Eczema (AE). Interestingly, both AMD and AE are characterized by the alteration of immune/inflammatory pathways similar to Ps and PsA [13,14].

In addition, a secondary aim of the study was the investigation of polymorphisms in miRNA-encoding genes, which may be associated with the susceptibility to Ps and/or PsA through the epigenetic regulation of inflammatory and cell proliferation pathways. On this subject, a polymorphism (rs2910164, C/G) located in the *MicroRNA 146a* (*MIR146A*, 5q33.3) gene was selected as a potential susceptibility biomarker in Ps and/or PsA. In fact, the *MIR146A* gene codes for homonymous miRNAs (miR-146a-5p and miR-146a-3p), which are known to take part in inflammatory and cell proliferation pathways and have been found associated with several multifactorial disorders (such as cancer, Alzheimer’s Disease, and AMD). Given these data, the rs2910164 variant has been selected considering that it has been found to contribute to these disorders [15].

## 2. Results

**Figure 1:**
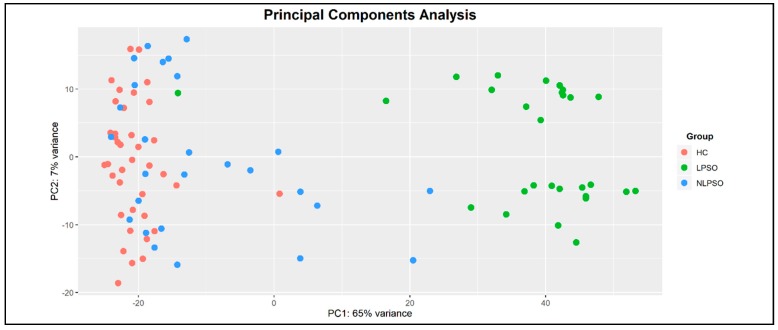
Principal component analysis (PCA) plot shows a substantial separation between transcriptomic profiles of psoriasis (Ps) lesional skin samples and healthy controls. Ps non-lesional skin samples showed a higher variability, although they were more similar to healthy controls compared to Ps lesional skin samples. These data have been extracted from the RNA-seq dataset published by Tsoi et al., 2019 [16]. HC: Healthy Controls; LPSO: Lesional Psoriatic Skin; NLPSO: Non-lesional Psoriatic Skin.

**Figure 2:**
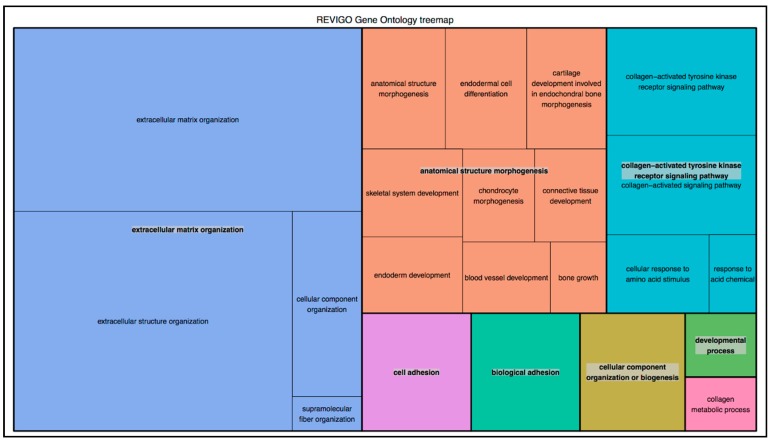
Tree-map of pathways clustered by semantic similarity from the set of collagen Differentially Expressed Genes (DEGs). The plot shows three super-clusters, namely Extracellular Matrix Organization, Anatomical Structure Morphogenesis, Collagen-activated Tyrosine Kinase Receptor Signaling Pathway and several clusters, such as Cell Adhesion and Collagen Metabolic Process. The prioritization of the three genes of interest followed the interpretation of this functional analysis. The size of the rectangles reflects the absolute log_10_*p*. These data have been extracted from the RNA-seq dataset published by Tsoi et al., 2019 [16].

Collagen genes were prioritized following the DEG and principal component analysis (PCA) analyses performed on the GEO dataset of Ps lesional skin samples versus healthy controls (Figure 1). Among the prioritized collagen genes, we found 28 DEGs that were subjected to GSEA on g:Profiler. The GSEA reported 55 BP-GOs terms, which, subsequently, were clusterized by semantic similarity through Revigo in order to facilitate their interpretation and the identification of relevant functional patterns (Figure 2).

Therefore, *COL6A5, COL8A1* and *COL10A1* were finally prioritized, considering their role in cell adhesion (GO:0007155, log_10_*p* = −8.7254), blood vessel development (GO:0001568, log_10_*p* = −4.2408) and skeletal system development (GO:0001501, log_10_*p* = −5.9813), respectively. Interestingly, these genes were found to be slightly downregulated in the psoriatic lesional skin samples compared to healthy controls (*COL6A5* Log2FC ± lfcSE: −1.03 ± 0.29; adj-*p*: 9.78 × 10^−4^; *COL8A1* Log2FC ± lfcSE: −1.64 ± 0.23, adj-*p*: 6.63 × 10^−12^; *COL10A1* Log2FC ± lfcSE: −0.64 ± 0.17 adj-*p*: 5.49 × 10^−4^). Given these preliminary data, three SNPs in the prioritized collagen genes (rs12488457, A/C, *COL6A5*; rs13081855, G/T, *COL8A1*; rs3812111, A/T, *COL10A1*) were subjected to genotyping analysis in order to test them as susceptibility biomarkers for Ps and/or PsA. Moreover, the genotyping analysis also included the SNP rs2910164 (C/G, *MIR146A*).

The obtained results were subjected to biostatistical and bioinformatic analysis in order to assess the possible association with the diseases of interest.

In particular, the differential frequency distribution (Table 1) and biostatistical analysis (Table 2) among the cases and the Italian controls revealed a positive association of rs12488457 (A/C, *COL6A5*) with both Ps and PsA. Similarly, a genetic association with both disorders has been found also for rs13081855 (G/T, *COL8A1*) (Table 2). Considering that *COL8A1* (3q12.1) and *COL6A5* (3q22.1) genes are both located on the long arm of chromosome 3, Linkage Disequilibrium (LD) analysis was performed in order to test the existence of LD between the SNPs in our cohorts of interest. As expected, the LD was excluded (Ps cohort: D’ = 0.231, r^2^ = 0.003, PsA cohort: D’ = 0.075, r^2^ = 0.001; Controls cohort: D’ = 0.092, r^2^ = 0.001), proving that the association of rs12488457 (*COL6A5*) and rs13081855 (*COL8A1*) was completely independent. Concerning the bioinformatic evaluation of the missense SNP rs12488457 (*COL6A5*), interrogation of Mutation Taster, Human Splicing Finder, Polyphen-2 and ExPaSy-ProSite showed a strong impact of the variant, which could potentially affect protein structure [14]. The analysis performed by Mutation Taster and Human Splicing Finder on the intronic rs13081855 (*COL8A1*) SNP revealed that the variant does not affect the splicing. The biostatistical analysis performed on the results obtained by rs3812111 (*COL10A1*) genotyping unveiled an exclusive association with PsA but not with Ps, suggesting thereby that the SNP could represent a potential differential biomarker between Ps and PsA (Table 2). Moreover, the bioinformatic analysis on this intronic variant by Mutation Taster and Human Splicing Finder did not report significant splicing changes for this SNP compared to the wild-type sequence. However, the rs3812111 was found to be in total LD (D’ = 1, r^2^ = 1) with the rs1064583 (A/G, *COL10A1*) in the Utah Residents (CEPH) with Northern and Western European Ancestry (CEU) population reported in the 1000Genomes database. Given this information, we evaluated the functional effect of this SNP by interrogating Mutation Taster, Polyphen-2 and Human Splicing Finder. In particular, the SNP rs1064583 is a missense methionine/threonine located in the exon 3 of the gene. According to Mutation Taster, the aminoacidic change has not been predicted to significantly affect the protein structure. Accordingly, Polyphen-2 reported a score of 0, consistent with a very low impact of the variant. Human Splicing Finder, instead, reported that the SNP may alter a regulatory splicing site.

Concerning rs2910164 (C/G, *MIR146A*), the biostatistical results supported a positive association with Ps and PsA (Table 2). *MIR146A* encodes two miRNAs, hsa-miR-146a-5p and hsa-miR-146a-3p, which are involved in the post-transcriptional regulation of several genes. On this subject, the bioinformatic analysis unveiled that the G allele may disrupt the interaction of miR-146a-3p with *Interleukin 13* (*IL13*, 5q31.1), *Tumor Necrosis Factor-Alpha-Induced Protein 3* (*TNFAIP3*, 6q23.3), *Serine/Threonine protein Kinase 40* (*STK40*, 1p34.3) and *KIF3A*, as well as it is able to create new binding sites for *Collagen Type IV Alpha-3* (*COL4A3*, 2q36.3) and *Late Cornified Envelope Protein 3D* (*LCE3D*, 1q21.3) (Table 3).

Overall, these results showed that rs12488457 (*COL6A5*), rs13081855 (*COL8A1*) and rs2910164 (*MIR146A*) represent susceptibility variants shared between Ps and PsA. rs3812111 (*COL10A1*), instead, is a risk variant specific for PsA.

## 3. Discussion

This study investigated the SNPs rs12488457 (A/C, *COL6A5*), rs13081855 (G/T, *COL8A1*), rs3812111 (A/T, *COL10A1*) and rs2910164 (C/G, *MIR146A*) as potential susceptibility biomarkers for Ps and/or PsA. The collagen genes were prioritized performing an in silico DEG analysis and GSEA on RNA-seq data of skin samples of Ps and healthy control subjects. In addition, the SNPs within these genes were selected considering that they have been investigated as susceptibility factors for complex diseases for which the alteration of immune-inflammatory processes is known to play a role in the pathogenesis [13,14,15].

The biostatistical analyses of the genotyping data unveiled intriguing results. In particular, rs12488457 (A/C, *COL6A5*) was significantly associated with both diseases (Table 2). This SNP is located in the 9th exon of the *COL6A5* gene and causes an amino acid substitution from threonine to proline at the 1280th position of the resulting protein. Bioinformatic analysis reported that the SNP might alter the canonical von Willebrand factor A7 domain (VWFA7) and nonhelical domains of the resulting protein product. Moreover, the SNP has been predicted to affect the splicing regulatory mechanisms and consequently, potentially hamper protein function. *COL6A5* codes for the α5 chain of Collagen VI, which is mainly expressed in the dermal papillae within the dermo-epidermal junctions, where it is involved in the cellular adhesion processes and in the ECM-cell interactions [14]. Moreover, the bioinformatic analysis performed on David, KEGG, g:Profiler and Revigo together with literature data [17] unveiled that Collagen VI and, in particular, the COL6A5 protein may take part in focal adhesion (KEGG:04510), phosphatidylinositol-3-kinase (PI3K)/AKT Serine/Threonine Kinase (AKT) (KEGG:04151) pathways and, thus, influence cell survival and proliferation. These data therefore support the potential contribution of *COL6A5* to the etiopathogenesis of Ps and PsA. Interestingly, rs12488457 (*COL6A5*) was found to be not significantly associated with AE in a previous work performed on a large cohort of patients of Mediterranean origin [14], suggesting that it may represent a risk variant specific for Ps and PsA.

rs13081855 (G/T, *COL8A1*) has been associated with a higher risk of Ps and PsA in our cohort (Table 2). This SNP is an intronic variant within the *COL8A1* gene, encoding the α1 chain of Collagen VIII. The bioinformatic analysis did not report any potential alteration of splicing mechanisms for this variant. The interrogation of g:Profiler showed that the COL8A1 protein is involved in ECM remodeling, maintenance of the structure of blood vessels and in neovascularization. In fact, COL8A1 is known to interact with the proangiogenic factor VEGFA [13]. Moreover, this gene has been found to contribute to neovascular AMD susceptibility [13,18], which is mainly characterized by an aberrant growth of new vessels in the retinal tissue. Of note, it is known that psoriatic patients may experience an aberrant vascularization of the dermis towards the epidermis, due to an overexpression of proangiogenic factors induced by the alteration of cell proliferation pathways. These vessels are characterized by tortuous capillary loops, which may contribute to exacerbate the disease symptoms in patients affected by Ps and PsA [19,20].

Consistent with these findings, rs13081855 (*COL8A1*) has been also associated with susceptibility to AE, suggesting that this SNP could represent a risk variant associated with inflammatory and immune-mediated complex disorders. Further research will be useful to explore if alterations in the expression and structure of Collagen VIII could exert a role in the psoriatic-related aberrant neovascularization.

Concerning rs3812111 (*COL10A1*), this study showed an association with a higher risk to develop PsA, but not Ps (Table 2). This SNP is an intronic variant, located in the *COL10A1* gene that codes for the α1 chain of Collagen X. As for rs13081855, bioinformatic analysis on this SNP did not report any potential alteration of splicing. However, rs3812111 has been found in total LD with an exonic SNP located in the same gene (*COL10A1*), namely rs1064583, which has been predicted to cause splicing changes. Therefore, rs1064583 will be included in further studies to be better characterized in affected patients and thus evaluate its potential association with susceptibility to PsA. The bioinformatic functional analysis revealed that *COL10A1* plays a role in maintaining bone homeostasis and cartilage metabolism as well as in the organization and remodeling of ECM. This result supports the potential implication of bone and cartilage metabolism-related processes in the onset and progression of PsA [20]. Accordingly, it has been found that Collagen X metabolism was enhanced in patients affected by PsA and Axial Spondyloarthritis [21]. This evidence supports *COL10A1* as a contributor to the rheumatological features observed in PsA. The present study is in line with the specific association of variants in *IL4* and *KIF3A* with PsA but not with Ps [7]. As a matter of fact, both *IL4* and *KIF3A* have been found to be implicated in bone metabolism as well, thus supporting that the network of genes regulating the metabolism and homeostasis of bone may take part in the etiopathogenesis of PsA. However, the real involvement of bone metabolism in PsA is still unclear and needs further investigation.

Finally, a significant association of rs2910164 (C/G, *MIR146A*) has been found with both Ps and PsA (Table 2). This variant is located within the miRNA seed region of miR-146a, encoded by the *MIR146A* gene. Several studies tried to investigate the association of this SNP with Ps, but results were conflicting [22]. In the present study, the G allele confers a higher susceptibility to Ps and PsA. Indeed, this allele results to be the most frequent allele in the European general population according to the GnomAD database (77%, Table 1). Interestingly, the prevalence of Ps in the European population is higher (3–4%) compared to the prevalence observed in the Chinese Han population (<1%), in which the C allele of the rs2910164 SNP is the most frequent (Table 1) [23]. This evidence supports the potential contribution of the rs2910164 G allele to disease susceptibility together with other predisposing factors.

Concerning the effect of this variant on miRNA function, the bioinformatic predictive analysis on PolymiRTs unveiled that the rs2910164 G allele may alter the binding of miR-146a to its targets by disrupting canonical sites or creating new ones (Table 3). In particular, the G risk allele has been predicted to disrupt the binding site for *IL13*, *TNFAIP3*, *STK40* and *KIF3A*. Intriguingly, *IL13* and *TNFAIP3* code for proteins involved in the inflammatory-related cytokine signaling and both of them have been previously associated with Ps [6]. Moreover *IL13* has been also highlighted as a susceptibility marker for PsA in individuals already suffering from Ps [24]. As previously discussed, *KIF3A* is involved in bone metabolism and has been associated with PsA, but not with Ps. The functions of *STK40* are still unclear, although a role for this gene in the regulation of keratinocyte proliferation and hair follicle differentiation has been described [25]. Therefore, the presence of the risk allele (rs2910164 G) in the miRNA sequence may cause downstream deregulations that exacerbate the inflammatory context and influence the proliferation-related processes, contributing to the etiopathogenesis of Ps and PsA.

Moreover, this variant has been predicted to make miR-146a able to target *COL4A3* and *LCE3D*. Although *COL4A3* has not been characterized in psoriatic diseases, its molecular function within the ECM (Table 3) and tissue localization (within the basement membrane of the skin) suggest that it may be interesting to further investigate the role of this gene in skin-related disorders [26]. *LCE3D* contributes to the formation of the cornified envelope of the stratum corneum and the maintenance of the barrier function of the epidermis. Of note, *LCE3D* has been found to be associated with Ps only in the Chinese population up to date [27]. Therefore, the downregulation of these genes targeted by the *MIR146A* variant may contribute to the dysfunction of epidermal homeostasis in Ps and PsA. All these in silico analyses highlight the need of validation of these targets by in vitro experiments in order to investigate the functional effects of the alterations of miR-146a in the psoriatic context. Moreover, the expression levels and the functions of miR-146a should be deeper explored.

rs2910164 (*MIR146A*) may influence not only the binding affinity of the target mRNA but also the expression of *MIR146A*, by affecting the pre-miRNA stability. Of note, the previously performed bioinformatic analysis revealed that the G allele of this variant may enhance the stability of the pre-miRNA secondary structure, leading to a potential improvement of the miRNA maturation process and thus to an enhanced production of the resulting miRNAs [15]. This evidence is line with literature data showing an overexpression of *MIR146A* in Ps [22,28]. However, miR-146a has also been regarded as a negative regulator of inflammation, because it is able to target *Interleukin 1 Receptor-Associated Kinase 1* (*IRAK1*, Xq28) and *TNF Receptor-Associated Factor 6* (*TRAF6*, 11p12), that are well-known pro-inflammatory factors [29,30].

Thus, the expression of miR-146a in psoriatic skin [23] needs to be further investigated because it may represent a protective mechanism against inflammation, although it may also activate feedback mechanisms that ultimately lead to the exacerbation of the inflammatory response. Moreover, it would be interesting to expand the analysis of genetic variants within other miRNAs able to influence Ps/PsA-related signaling pathways.

**Figure 3:**
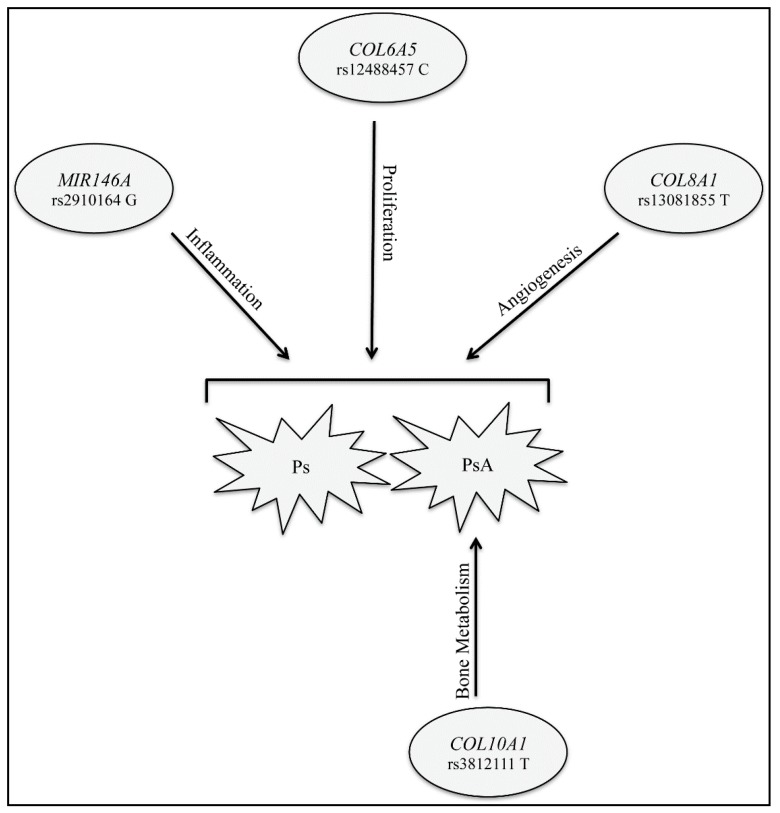
*MIR146A, COL6A5, COL8A1* contribute to the susceptibility to both Ps and PsA, with an effect on the inflammation, proliferation and angiogenesis processes, respectively. *COL10A1* is involved in the etiopathogenesis of PsA through its role in bone metabolism.

In conclusion, the present study highlighted *COL6A5*, *COL8A1* and *MIR146A* as new potential susceptibility biomarkers for Ps and PsA. This shared association reflects the involvement of common mechanisms involved in their onset and progression. In particular, *COL6A5* may take part in proliferation-related pathways and *COL8A1* may exert a role in angiogenic networks, both of which can be altered in Ps/PsA and lead to the activation of inflammatory processes, in which both products of *MIR146A* are involved (Figure 3). This evidence needs to be confirmed by functional studies. On the other hand, *COL10A1* could be utilized as a differential biomarker between Ps and PsA (Figure 3). This differential association, together with the study of other bone-related genes, [7] may explain the deregulation of bone metabolism in the etiopathogenesis of PsA.

Indeed, the diagnostic utility of the associated variants needs to be evaluated upon validation and further investigation on larger cohorts, and may be implemented to include the analysis of other genetic and epigenetic potential contributors, such as known genetic risk factors [6,7], collagen-coding and noncoding RNAs genes. In fact, the results described in the present study could be useful for setting up innovative and personalized treatments [31] aimed to counteract the development and the progression of Ps and PsA.

## 4. Materials and Methods

### 4.1. Prioritization of Collagen Genes

The prioritization of the collagen genes was based on data from a RNA-seq study [16], whose dataset is available on the GEO database (GSE121212). A DEGs analysis was performed on a subset of samples, including lesional and nonlesional skin samples from 28 Ps patients and 38 healthy controls.

This analysis was conducted using the R-software package DESeq [32,33]. Principal component analysis (PCA) was performed to visualize differences between sample groups (Figure 1). In particular, the PCA plot was generated from the counts data by means of Variance Stabilizing Transformation (VST) included in DESeq. For each comparison, the base means across samples, the log^2^ Fold Changes (Log2FC), the standard errors (lfcSE), test statistics, *p*-values (*p*) and adjusted *p*-values (adj-*p*) through Benjamini–Hochberg correction, were calculated. Subsequently, a GSEA was performed using g:Profiler [34] on a list of collagen DEGs with an adj-*p* < 0.05 extracted from the comparison of Ps lesional skin samples versus healthy controls. Moreover, the prioritization was also performed considering the most relevant biological functions through a semantic clusterization of Biological processes-Gene Ontologies (BP-GO) terms performed by Revigo [35].

### 4.2. Study Cohorts

The study was performed on 1417 Italian subjects, which included 393 patients with Ps, 424 patients with PsA and 600 healthy individuals. All of them were recruited from “Tor Vergata” General Hospital of Rome. The recruited patients with Ps and PsA had an average age of 57 ± 15 and 62 ± 14 years, respectively. The male/female ratio was 62:38. Healthy subjects were matched according to age and gender of the patient groups. The clinical diagnosis was assessed by expert dermatologists and rheumatologists and fulfilled the classical clinical criteria and the CASPAR Study Group criteria [36]. In particular, Ps patients were characterized by moderate-to-severe skin lesions throughout the body. Patients with PsA were affected not only by psoriatic lesions localized on the skin, nails and scalp, but also by peripheral arthritis in the elbows, hips and knees; dactylitis and enthesitis with axial involvement. Moreover, all these patients were negative for rheumatoid factor. The diagnosis of Ps, PsA and other immune-mediated diseases was excluded for all control subjects at the time of recruitment. The research was approved by the Ethics Committee of “Tor Vergata” General Hospital of Rome (reference number: 149/19, approved on 18 September 2019)) and was performed according to the Declaration of Helsinki. All participants provided signed informed consent.

### 4.3. DNA Extraction and Genotyping Analysis

The DNA of patients and control subjects was extracted from peripheral blood samples. The extraction was performed using the EZ1 Advanced XL automated extractor and the EZ1 DNA Blood 200 μL Kit (Qiagen, Valencia, CA, USA). The genotyping analysis was performed by Real Time PCR on a 7500 Fast Real Time PCR (Applied Biosystems, Warrington, UK) instrument according to manufacturer’s instructions. Specific predesigned TaqMan assays were utilized for *COL6A5* (rs12488457, A/C), *COL8A1* (rs13081855, G/T), *COL10A1* (rs3812111, A/T) and *MIR146A* (rs2910164, C/G) genotyping. The results were interpreted using Sequence Detection System 2.1 software (Applied Biosystems, Warrington, UK).

Each Real Time PCR run was performed using a negative control and three positive control samples previously confirmed by Sanger sequencing (BigDye Terminator v3.1, BigDyeXTerminator, Applied Biosystems, Warrington, UK) on ABI3130xl (Applied Biosystems, Warrington, UK). Direct sequencing of the samples was conducted following the manufacturer’s indications, as well.

### 4.4. Biostatistical Analysis

The genotyping results were subjected to biostatistical analysis to evaluate the genetic association with Ps and PsA. Allele and genotype frequencies of the analyzed SNPs were calculated by direct counting. Hardy–Weinberg Equilibrium (HWE) was tested at each locus by comparing the observed genotype frequencies with those expected under HWE. The resulting data were considered in HWE with *p* > 0.05. The association of the SNPs was measured by calculating the *p* through a contingency table and the χ^2^ test. The cut-off for statistical significance was set at *p* < 0.05. The strength of association was determined by calculating the Odds Ratio (OR) and considering a 95% confidence interval. All statistical analyses were performed using the SPSS program, ver. 23 (IBM Corp, Armonk, NY, USA).

### 4.5. Bioinformatic Analysis

Bioinformatic tools were employed to assess the in silico functional role of the analyzed SNPs [13,14]. MutationTaster [37], Polyphen-2 [38], ExPaSy-Prosite [39] were used to predict the impact of the SNPs on protein structure and function. Human Splicing Finder [40] was interrogated to assess whether the variants may alter the splicing activity. All of these tools exploit algorithms to calculate a score and predict the effect size of the variants of interest. The functional effect of the miRNA variant rs2910164 was assessed by the interrogation of PolymiRTs database 3.0 (University of Tennessee Health Science Center, Memphis, TN, USA), that annotates genetic variants within miRNA seed regions and 3′UTR of target genes and predicts the consequence of the variants on the interactions among miRNAs and target mRNAs [41]. The Linkage Disequilibrium (LD) pattern analysis was performed on the Haploview tool, which evaluates the LD patterns and performs haplotype block analysis, providing the pairwise LD plot with the results displayed in color scheme [42].

## Figures and Tables

**Table 1:** Allele frequencies of the SNPs of interest in Ps, PsA, Italian healthy control cohorts and across different populations. The AMR, EAS, EUR and AFR allele frequencies for rs12488457, rs13081855 and rs3812111 have been extracted from 1000 Genomes, whereas the rs2910164 frequencies are referred to GnomAD. * These frequencies refer to the Non-Finnish European (NFE) Population. AMR: American; EAS: East Asian; EUR: European; AFR: African.

Gene	SNP	Allele Frequencies (Ps)	Allele Frequencies (PsA)	Allele Frequencies (Italian Controls)	AMR Allele Frequencies	EAS Allele Frequencies	EUR Allele Frequencies	AFR Allele Frequencies
***COL6A5***	rs12488457 A/C	A: 0.31	A: 0.35	A: 0.43	A: 0.57	A: 0.83	A: 0.28	A: 0.95
		C: 0.69	C: 0.65	C: 0.57	C: 0.43	C: 0.17	C: 0.72	C: 0.05
***COL8A1***	rs13081855 G/T	G: 0.90	G: 0.89	G: 0.95	G: 0.95	G: 0.95	G: 0.90	G: 0.97
		T: 0.10	T: 0.11	T: 0.05	T: 0.05	T: 0.05	T: 0.10	T: 0.03
***COL10A1***	rs3812111 A/T	A: 0.37	A: 0.34	A: 0.40	A: 0.33	A: 0.26	A: 0.40	A: 0.74
		T: 0.63	T: 0.66	T: 0.60	T: 0.67	T: 0.74	T: 0.60	T: 0.26
***MIR146A***	rs2910164 C/G	C: 0.26	C: 0.25	C: 0.30	C: 0.31	C: 0.63	* C: 0.23	C: 0.40
		G: 0.74	G: 0.75	G: 0.70	G: 0.69	G: 0.37	* G: 0.77	G: 0.60

**Table 2:** Biostatistical results obtained by the genotyping analysis on patients with Ps, PsA and healthy control subjects. ns: not significant.

Gene	SNP	Disease	*p*	OR (95% C.I.)
***COL6A5***	rs12488457 A/C	Ps	1.39 × 10^−8^	C: 1.74 (1.43–2.10)
		PsA	5.12 × 10^−5^	C: 1.46 (1.21–1.75)
***COL8A1***	rs13081855 G/T	Ps	4.52 × 10^−4^	T: 1.85 (1.31–2.64)
		PsA	1.19 × 10^−6^	T: 2.24 (1.61–3.12)
***COL10A1***	rs3812111 A/T	Ps	ns	-
		PsA	0.005	T: 1.30 (1.08–1.56)
***MIR146A***	rs2910164 C/G	Ps	0.04	G: 1.23 (1.00–1.51)
		PsA	0.01	G: 1.29 (1.04–1.61)

**Table 3:** Predicted effects of the rs2910164 C/G variant on the binding activity of *MIR146A* (miR-146a-3p) and the molecular functions of target genes potentially related to Ps/PsA.

Gene	SNP	Effect Allele	Predicted Functional Effect	Target Gene	Gene Molecular Function
***MIR146A***	rs2910164 C/G	G	Target sites disrupted	*IL13*	Cytokine signaling
				*TNFAIP3*	Cytokine signaling
				*STK40*	Keratinocyte proliferation
				*KIF3A*	Bone metabolism
			Target sites created	*COL4A3*	ECM remodeling
				*LCE3D*	Epidermal barrier
